# A Good Case of Recurrent Pneumonia

**DOI:** 10.1177/2324709618802869

**Published:** 2018-09-29

**Authors:** Ankit Bhargava, Rachel Eisenstadt, Jennifer A. Shih, Viranuj Sueblinvong

**Affiliations:** 1 Emory University School of Medicine, Atlanta, GA, USA

**Keywords:** Good’s syndrome, *Bordetella bronchiseptica*, *Pneumocystis jirovecii*, recurrent pneumonia

## Abstract

*Bordetella bronchiseptica* infection is a common cause of pneumonia in animals but rarely causes disease in humans. Additionally, coinfection with *Pneumocystis jirovecii* is very uncommon and is occasionally seen in patients with acquired immunodeficiency syndrome (AIDS). We report a case of a 61-year-old HIV-negative man, who presented with hypoxic respiratory failure 2 days after completion of systemic intravenous antibiotic treatment for *B bronchiseptica.* His past medical history was significant for a benign thymoma. The patient was found to be coinfected with *B bronchiseptica* and *P jirovecii.* Laboratory results showed panhypogammaglobulinemia and low absolute B- and CD4 T-cells. Therefore, the patient was diagnosed with Good’s syndrome. However, despite treatment with intravenous antibiotics and intravenous immunoglobulin, the patient continued to deteriorate and expired. This patient demonstrates the importance of recognizing this rare immunodeficiency early in order to improve morbidity and mortality. Furthermore, this case highlights the importance of early immunoglobulin screening in the presence of asymptomatic thymoma.

## Introduction

Good’s syndrome is a rare entity that causes B- and T-cell immunodeficiency in adults, leading to an increase in susceptibility to encapsulated organisms, fungal, and opportunistic infections (OIs).^1^ This case highlights the importance of maintaining a high clinical suspicion for uncommon immunodeficiency conditions in patients who present with unusual combinations of infection.^2^ Appropriate workup to obtain a diagnosis will allow for timely delivery of appropriate treatment.

## Case Report

**Figure 1.**
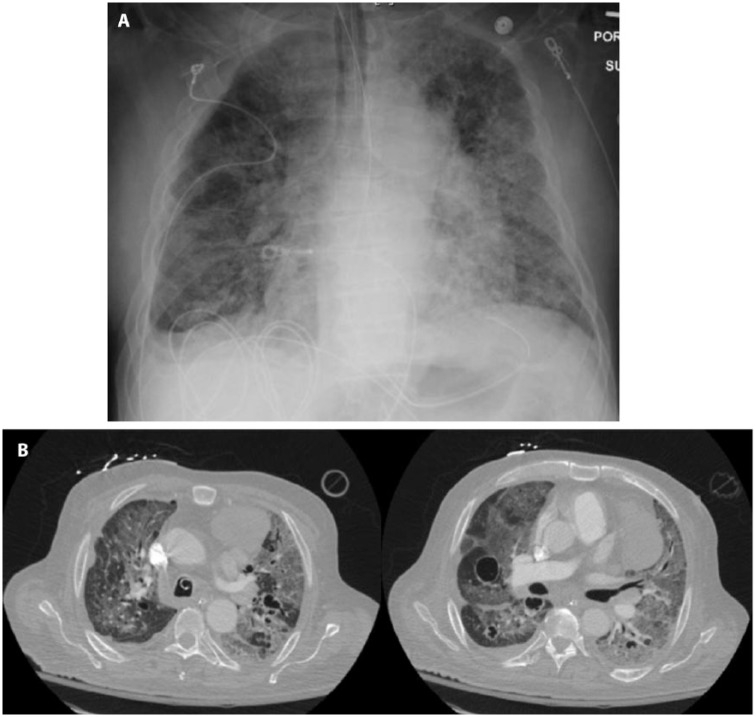
Chest radiographic findings at the time of hospital admission. (A) Chest X-ray showing endotracheal tube and diffuse ground glass opacity and (B) representative cut of chest computed tomography scan showing bilateral ground glass opacity and cavitary lesions.

A 61-year-old homeless man with a past medical history significant for benign spindle-cell thymoma presented with acute hypoxic respiratory failure. Two months prior, he was treated for *Bordetella bronchiseptica* pneumonia and empyema with intravenous (IV) antibiotics and right pleural decortication. Evaluation during the first hospitalization was negative for HIV, hepatitis B infection, syphilis, blastomycosis, and coccidioidomycosis. He was discharged but was subsequently rehospitalized within 1 week with recurrent pneumonia. During this second hospitalization, he was treated with another 2-week course of antibiotics with some improvement in symptoms and was discharged home. Two days after being discharged, he presented to our institute with hypoxic respiratory failure requiring endotracheal intubation. Pertinent findings on physical examination were fever, hypoxia, and tachycardia. Oral candidiasis was noted. Lung auscultation revealed coarse and mechanical breath sounds bilaterally. Chest radiographic findings showed bilateral patchy airspace opacities (Figure 1A). Computed tomography scan of the chest showed a stable, large anterior mediastinal mass, multiple cavitary lesions, and diffuse ground-glass opacities (Figure 1B). The patient was started on broad-spectrum IV antibiotics with cefepime and vancomycin. Examination of the bronchoalveolar lavage revealed *B bronchiseptica* and *Pneumocystis jirovecii.* The patient’s antibiotics regimen was changed to piperacillin/tazobactam, sulfamethoxazole-trimethoprim with prednisone, and fluconazole. Repeat HIV serology was negative. Laboratory results showed panhypogammaglobulinemia and low total B- and CD4 T-cells (Table 1). IV immunoglobulin (IG) treatment (400 mg/kg every 3-4 weeks) was initiated. He was evaluated for possible thymectomy but was not a surgical candidate due to his clinical condition. His clinical status continued to deteriorate, and he subsequently suffered cardiac arrest and death.

**Table 1.** Laboratory Results.

Test	Result	Normal Range
Complete blood count		
Hemoglobin	9.9 (g/dL)	12.9-16.1 (g/dL)
White blood cell count	9.4 (×10^3^ cell/µL)	4.2-9.1 (×10^3^ cell/µL)
Neutrophil (%)	90	
Lymphocyte (%)	7	
Monocyte (%)	3	
Absolute neutrophil	8.36 (×10^3^ cell/µL)	0.67-6.41 (×10^3^ cell/µL)
Absolute lymphocyte	0.64 (×10^3^ cell/µL)	0.72-3.29 (×10^3^ cell/µL)
Absolute monocyte	0.31 (×10^3^ cell/µL)	0.14-0.71 (×10^3^ cell/µL)
Platelet count	302 (×10^3^ cell/µL)	150-400 (×10^3^ cell/µL)
Immunophenotype		
Absolute B cell (CD19)	<1 (cell/µL)	91-610 (cell/µL)
CD4	203 (cell/µL)	430-1800 (cell/µL)
Immunoglobulin (Ig)		
Total IgG	367	620-1400 mg/dL^a^
Total IgM	11	45-250 mg/dL^a^
Total IgA	60	80-350 mg/dL^a^
Total IgE	<1	<1 mg/dL^a^

a Depicts a normal value in patients without acute infection.

## Discussion

The eponymous Dr Good first described the association of thymoma, hypogammaglobulinemia, and increased susceptibility to OI in 1954. Good’s syndrome has since been classified as an adult-onset immune deficiency with low to absent B-cells, derangement in cell-mediated immunity (CD8:CD4 imbalance, low CD4 count), and thymoma, without formal diagnostic criteria.^3^ The etiology of this immune dysfunction remains elusive. Good’s syndrome is distinct from common variable immunodeficiency in the presence of both hypogammaglobulinemia and reduced B-cell populations. This disease process clarifies the role of the thymus both in educating T-cells and producing an appropriate response in B-cells. Two proposed theories for the etiology of associated hypogammaglobulinemia in patients with thymoma, including bone marrow suppression and paraneoplastic phenomena, supported by the association with pure red cell aplasia.^4,5^

While relatively rare, the incidence among patients with thymoma and hypogammaglobulinemia may be as high as 6% to 11%.^4^ A mean age at the time of diagnosis is 59.1 years (12-90 years).^6^ In 2.4% of patients who first presented with thymoma, 37.9% presented with both thymoma and infection, whereas only 19.7% presented with infection.^7^ Autoimmunity is a common phenomenon in patients with Good’s syndrome. About 58.6% of patients have a secondary autoimmune condition, most commonly pure red cell aplasia (34.8%), myasthenia gravis (15.7%), and oral lichen planus (12.4%).^5,8,9^ The clinical course of Good’s syndrome has been reported to be more severe when compared with common variable immunodeficiency, with a 10-year survival of 33% versus 95%, respectively.^7^ Interestingly, T-cell count does not accurately correlate with associated risk of OI for patients with Good’s syndrome. Chest radiography can miss thymoma in up to 25% of patients.^10^ Due to deficiency of B- and T-cells, patients with Good’s syndrome are susceptible to a variety of infections, including encapsulated bacteria (ie, *Haemophilus, Streptococcus, Pseudomonas, Klebsiella, Bordetella*), fungi (ie, *Candida, Pneumocystis jirovecii*), viral infections, and protozoa (ie, *Giardia*).^7,11^ Diagnosis should be made through radiologic evaluation of thymoma, measurement of IG serum levels, phenotyping of lymphocytes, evaluation for B-cell population depletion, and CD8:CD4 T-cell derangement.^4^ Specific treatment of Good’s syndrome includes high-dose IVIG (400 mg/kg IV every 3-4 weeks) to improve humoral immune response and to help prevent life-threatening OIs. Thymectomy is recommended to prevent other immunological manifestations of thymoma including myasthenia gravis, pure red cell aplasia, and pernicious anemia.^3,10^ However, several reports suggest that hypogammaglobulinemia persists following thymectomy, and patients with Good’s syndrome remain at risk for OI.^12,13^

Our patient’s presentation demonstrates the importance of early recognition of this rare immunodeficiency.^2^ A delay in the diagnosis may result in increased morbidity and mortality. This case highlights the importance of early reflexive IG screening, even in the presence of asymptomatic thymoma. In the presence of unexplained OI alongside history of thymoma, a high suspicion for acquired immune deficiency may allow for expedient delivery of appropriate therapy.

In summary, Good’s syndrome should be suspected in patients present with uncommon encapsulated infection (ie, *Bordetella* spp) and OIs. IVIG should be administered to prevent life-threatening OIs. Reflexive immunophenotyping and IG levels in patients with asymptomatic thymomas may allow early diagnosis of Good’s syndrome and early implementation of treatment.
